# Association of Insomnia, Sleep Quality, and Sleep Duration With Risk of Physical Frailty in Middle-aged and Older People With HIV

**DOI:** 10.1093/ofid/ofad566

**Published:** 2023-11-09

**Authors:** Qionggui Zhou, Yingying Ding, Xiaoxiao Chen, Shanling Wang, Haijiang Lin, Na He

**Affiliations:** Department of Epidemiology, School of Public Health, and Key Laboratory of Public Health Safety of Ministry of Education, Fudan University, Shanghai, China; Yi-Wu Research Institute, Fudan University, Shanghai, China; Shanghai Institute of Infectious Diseases and Biosecurity, Fudan University, Shanghai, China; Department of Epidemiology, School of Public Health, and Key Laboratory of Public Health Safety of Ministry of Education, Fudan University, Shanghai, China; Yi-Wu Research Institute, Fudan University, Shanghai, China; Department of Epidemiology, School of Public Health, and Key Laboratory of Public Health Safety of Ministry of Education, Fudan University, Shanghai, China; Taizhou City Center for Disease Control and Prevention, Taizhou, Zhejiang Province, China; Taizhou City Center for Disease Control and Prevention, Taizhou, Zhejiang Province, China; Department of Epidemiology, School of Public Health, and Key Laboratory of Public Health Safety of Ministry of Education, Fudan University, Shanghai, China; Taizhou City Center for Disease Control and Prevention, Taizhou, Zhejiang Province, China; Department of Epidemiology, School of Public Health, and Key Laboratory of Public Health Safety of Ministry of Education, Fudan University, Shanghai, China; Yi-Wu Research Institute, Fudan University, Shanghai, China; Shanghai Institute of Infectious Diseases and Biosecurity, Fudan University, Shanghai, China

**Keywords:** HIV, insomnia, physical frailty, sleep duration, sleep quality

## Abstract

**Background:**

Frailty is one of the major concerns among aging people with HIV (PWH). Evidence regarding the association between sleep disorders and physical frailty in PWH is limited.

**Methods:**

PWH and HIV-negative individuals aged ≥40 years were included and frequency-matched in a 1:2 ratio by sex and age. Logistic regression models were used to estimate the odds ratios (ORs) and 95% CIs of the association between sleep disorders and physical frailty, and restricted cubic splines were used to describe the dose–response association. The contribution of depression to the association was estimated by mediation analysis.

**Results:**

A total of 1526 PWH and 3052 HIV-negative individuals were included. Logistic regression indicated that insomnia (OR, 3.05; 95% CI, 1.63–5.72) and poor sleep quality (OR, 2.32; 95% CI, 1.21–4.45) were significantly associated with physical frailty in middle-aged and older PWH, especially in those with current CD4+ T-cell counts <350 cells/µL, but not in HIV-negative participants. A U-shaped and J-shaped dose–response relation between sleep duration and physical frailty was observed in PWH and HIV-negative participants, respectively. Shorter and longer sleep duration was associated with an increased risk of physical frailty in PWH. However, in HIV-negative participants, only longer sleep duration was associated with physical frailty. Mediation analysis revealed that depression mediated the relation between sleep disorders and frailty among PWH.

**Conclusions:**

Sleep disorders including insomnia, poor sleep quality, and short and long sleep duration were significantly associated with physical frailty among middle-aged and older PWH. Depression may play a mediating role in the sleep–frailty association.

As life expectancy has increased for people with HIV (PWH) receiving potent antiretroviral therapy (ART), the number of older PWH has expanded greatly [1, 2]. As PWH age, they experience an excess burden of non-AIDS comorbidities including polypharmacy, cognitive impairment, and frailty. Frailty is one of the geriatric syndromes characterized by increased vulnerability to stress due to cumulative decreases in the reserves of multiple physiological systems, leading to an increased risk of multiple adverse health outcomes such as falls, disability, hospitalization, and even mortality [3–5]. However, physical frailty may be prevented if treated appropriately under specific circumstances. Therefore, identifying modifiable risk factors related to physical frailty is important to prevent or delay the progression of physical frailty among PWH.

Sleep disorders are common and serious problems among middle-aged and older HIV-positive and HIV-negative individuals [6]. A meta-analysis of 27 studies including 9246 HIV-positive participants reported that the overall prevalence of sleep disorders was 58.0% in the HIV-positive population [7] compared with 30.5% in the HIV-negative population [8], suggesting that PWH suffered from a heavy burden of sleep disorders. Previous studies have found that sleep disorders including poor sleep quality, insomnia, nap duration, and either short or long sleep duration were significantly associated with physical frailty among HIV-negative participants [9–11]. However, studies on the association between sleep disorders and physical frailty remained scarce in middle-aged and older PWH. In addition, research has found that depression plays a mediating role between sleep quality and physical frailty in community-dwelling older adults [12]. However, to our knowledge, no study has specifically examined the mediating effect of depression on the association between sleep disorders and frailty among PWH.

Therefore, in this study, we aimed to investigate the associations of different symptoms of sleep disorders (insomnia, poor sleep quality, and sleep duration) with physical frailty and further examine whether depression mediated the association between sleep disorders and physical frailty in a population-based study of age/sex-matched middle-aged and older HIV-positive and HIV-negative individuals.

## METHODS

### Study Design and Participants

This cross-sectional study was derived from the baseline evaluation of the Comparative HIV and Aging Research in Taizhou (CHART) cohort, an ongoing prospective study of HIV and age-related comorbidities among PWH and HIV-negative individuals in Taizhou prefecture, Zhejiang province, China, from February 2017 to January 2020. The details of CHART have been described previously [13]. We enrolled 4895 participants aged ≥40 years, 1675 PWH who have registered with the national HIV/AIDS Comprehensive Response Information Management System (CRIMS) and 3220 HIV-negative individuals from 6 local communities in Taizhou during the same period.

For this study, we randomly selected HIV-positive and HIV-negative participants from the whole sample without missing data on physical frailty and frequency-matched them in a 1:2 ratio according to sex and 5-year age categories, using Proc SurveySelect in SAS. Ultimately, 4578 participants (1526 cases and 3052 controls) were included in the final analysis. The study was approved by the Institutional Review Board of Fudan University, Shanghai, China, with written informed consent was obtained from all participants.

### Data Collection and Assessments

Basic demographic and lifestyle characteristics of all participants were obtained at the baseline investigation of the CHART cohort by trained staff with a face-to-face questionnaire, including age, gender, smoking status, and alcohol use. HIV-related variables were extracted from CRIMS, including the date of HIV diagnosis, CD4 cell counts, CD8 cell counts, HIV RNA, ART regimens, etc. Nadir CD4+ T-cell count was defined as the lowest CD4+ T-cell count recorded.

Weight and height were measured twice with participants in light clothing, and the average was used in the analysis. Body mass index (BMI) was then calculated as weight divided by the square of height (kg/m^2^). Blood pressure (BP) was assessed twice after at least 5 minutes of rest, and the mean value was employed. Fasting blood samples were collected for glycated hemoglobin (HbA1c) and lipids measurement. Depressive symptoms were measured by the 9-item adapted version of the Zung Self-Rating Depression Scale [14, 15]. Neurocognitive performance was assessed using the Chinese version of the Mini-Mental State Examination (MMSE) [16].

Information on chronic conditions including hypertension, diabetes, dyslipidemia, obesity, and renal diseases was collected. Obesity was defined as BMI ≥28 kg/m^2^, according to the Working Group on Obesity in China criteria [17]. Hypertension was defined as systolic blood pressure (SBP) ≥140 mmHg or diastolic blood pressure (DBP) ≥90 mmHg or using antihypertensive drugs with previous clinical diagnosis. Diabetes was defined as HbA1c ≥6.5% or previous clinical diagnosis. Dyslipidemia was defined as total cholesterol ≥5.2 mmol/L, LDL cholesterol ≥3.4 mmol/L, or triglycerides ≥1.7 mmol/L. Chronic renal disease was defined as an estimated glomerular filtration rate (eGFR) <60 mL/min/1.73m^2^ or previous clinical diagnosis. Comorbidity was defined as the presence of ≥2 self-reported chronic diseases or conditions.

### Sleep Characteristics

The Pittsburgh Sleep Quality Index (PSQI) was used to measure subjective sleep quality and types of sleep disturbances in the previous month [18]. This scale consists of 19 items, including components of sleep quality, sleep latency, sleep duration, sleep efficiency, sleep disturbances, sleep medication use, and daytime dysfunction. Global scores provide an assessment of overall sleep quality, which ranges from 0 to 21, with a score >5 indicating poor sleep quality.

Information on sleep duration was collected by asking the following question, “During the past month, how many hours of actual sleep did you get at night (average hours for one night).” Sleep duration was divided into 5 groups: (short) <6 hours, 6–7 hours, 7–8 hours, 8–9 hours, and (long) ≥9 hours per night, with 7–8 hours per night as the reference category, according to recommendations from the National Sleep Foundation [19] and previous studies [20].

Insomnia symptoms were assessed by asking 4 sleep-related questions based on the Jenkins Sleep Problems Scale [21]: “Over the last month did you: 1) have trouble staying asleep, 2) have trouble falling asleep, 3) wake up too early and feel unable to get back to sleep, and 4) wake up several times per night and feel unable to get back to sleep?” Insomnia was defined as at least 1 question with an answer of “most nights” or “every night.”

### Physical Frailty

Physical frailty was assessed using the modified version of the Fried's Frailty Phenotype [22], which has been widely used among older PWH populations. The tool includes 5 components with self-report and objective measurements: unintentional weight loss, self-reported low physical activity, self-reported exhaustion, weak grip strength, and slow gait speed. Measurement details for each component have been published and described elsewhere [23]. Participants who met 0 components were classified as robust, 1 or 2 components as prefrailty, and 3 or more components as frailty.

### Statistical Analysis

Baseline characteristics of participants were summarized by HIV serostatus, with continuous data presented as median (interquartile range [IQR]) because of skewed distribution and categorical variables presented as number (%). Group comparisons were assessed using the χ² test, Fisher exact test, or Wilcoxon rank-sum test when appropriate.

Logistic regression models were used to estimate the odds ratios (ORs) and 95% CIs for the associations of insomnia, sleep quality, and sleep duration with risk of physical frailty by 3 models: model 1, unadjusted; model 2, adjusted for age, gender, smoking, alcohol consumption, depression, and neurocognitive impairment; and model 3, additionally adjusted for comorbidity and further adjusted for current CD4+ and CD8+ T-cell counts for PWH. Stratified analysis was performed among HIV-positive patients by CD4+ T-cell counts (≥350 cells/µL vs <350 cells/µL) [24]. To describe the dose–response association between sleep duration and frailty, we conducted restricted cubic splines in logistic regression, with 7.5 hours/night as the reference and 3 knots at the 10th, 50th, and 90th percentiles of sleep duration, adjusting for the same covariates as in model 3 above.

To examine whether depression played a mediating role in the association between sleep disorders (insomnia, sleep quality, sleep duration) and physical frailty, simple mediation analysis was conducted by the PROCESS procedure in R [25, 26], adjusting for the same covariates in the multivariate logistic regression model (model 3; except for depression). Sleep disorders were considered the independent variable (X), depression was the mediator variable (M), and physical frailty was the dependent variable (Y). The associations among the 3 variables are shown in Supplementary Figure 1. The indirect effects were calculated as the product of coefficients (a*b) and were estimated from 5000 bootstrap samples, representing the actual mediation [27]. Point estimates for the indirect effect were considered significant when the 95% CI did not include 0. Partial mediation was defined by the indirect effect (a*b) and direct effect (*c’*) both existing and pointing in the same direction, while full mediation was defined as the indirect effect (a*b) existing but no direct effect [27, 28].

All the analyses were conducted using SAS 9.4 (SAS Inst., Cary, NC, USA) and R 4.2.2 (R Foundation), with 2-sided *P* values <.05 considered statistically significant.

## RESULTS

### Participant Characteristics

**Figure 1.**
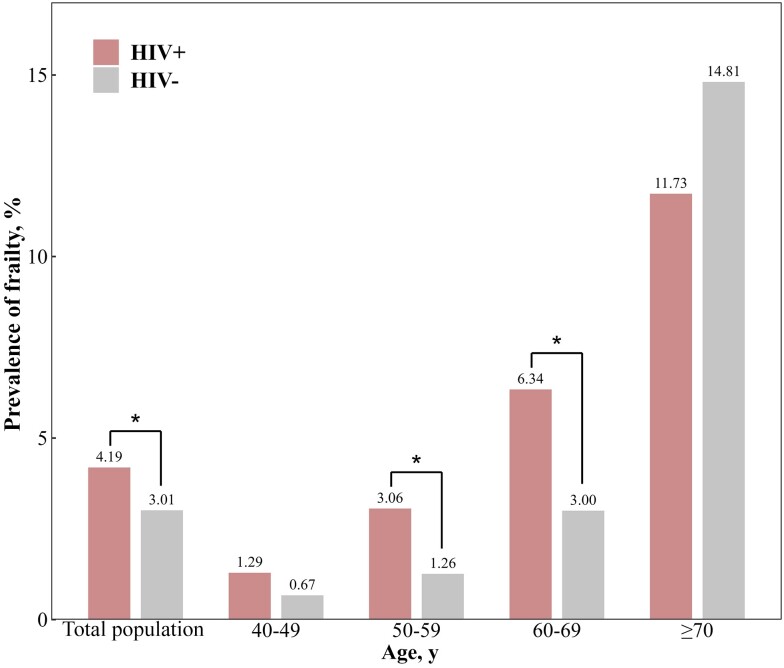
Prevalence of physical frailty across age groups in HIV-positive and HIV-negative participants. *P* values compare the proportion of frailty between HIV-positive and HIV-negative participants within 4 age groups (40–49 years, 50–59 years, 60–69 years, and ≥70 years) using the χ^2^ test or Fisher exact test when appropriate. *P < .05.

A total of 4578 participants, including 1526 HIV-positive (median age, 53.5 years; male 75.6%) and 3052 HIV-negative (median age, 53.3 years; male 75.6%), were included in the present analysis. Both groups were comparable by age and gender, but PWH included more individuals with neurocognitive impairment, depression, prolonged sleep duration, and chronic renal disease than HIV-negative individuals; and fewer individuals who reported smoking, alcohol consumption, hypertension, diabetes, dyslipidemia, and obesity (all *P* < .05) (Table 1). For the HIV-specific factors, the median current CD4+ T-cell count (IQR) was 352.0 (215.0–519.0) cells/µL, and the median duration since HIV diagnosis (IQR) was 1.0 (0.2–4.4) years. The prevalence of frailty in PWH increased in the 50–59 and 60–69 age groups but was lower in the ≥70 age group, compared with HIV-negative controls (Figure 1).

**Table 1.** Characteristics of Study Participants by HIV Serostatus

Characteristics	Total	HIV-Positive	HIV-Negative	*P* Value
	n = 4578	n = 1526	n = 3052	
Age, y	53.4 (46.4–62.7)	53.5 (46.3–62.9)	53.3 (46.5–62.6)	.994
Gender				1.000
Male	3459 (75.6)	1153 (75.6)	2306 (75.6)	
Female	1119 (24.4)	373 (24.4)	746 (24.4)	
Smoking				<.001
Never	2365 (51.7)	841 (55.1)	1524 (49.9)	<.001
Past	692 (15.1)	240 (15.7)	452 (14.8)	
Current	1521 (33.2)	445 (29.2)	1076 (35.3)	
Alcohol consumption	953 (20.8)	170 (11.1)	783 (25.7)	<.001
SBP, mmHg	129.0 (119.0–141.0)	126.0 (118.0–136.5)	130.5 (119.0–143.5)	<.001
DBP, mmHg	78.5 (71.5–85.5)	77.5 (70.5–84.0)	79.0 (72.0–86.5)	<.001
Total cholesterol, mmol/L	5.0 (4.4–5.6)	4.7 (4.1–5.4)	5.1 (4.5–5.8)	<.001
Triglycerides, mmol/L	1.8 (1.2–2.7)	1.7 (1.2–2.5)	1.9 (1.3–2.8)	<.001
LDL-C, mmol/L	2.7 (2.2–3.3)	2.5 (2.0–2.9)	2.9 (2.4–3.5)	<.001
HDL-C, mmol/L	1.1 (0.9–1.3)	1.0 (0.9–1.3)	1.1 (1.0–1.4)	<.001
Depression	744 (16.3)	496 (32.5)	248 (8.1)	<.001
Neurocognitive impairment	487 (10.6)	286 (18.7)	201 (6.6)	<.001
Insomnia	1189 (26.0)	403 (26.4)	786 (25.8)	.659
Poor sleep quality	1243 (27.2)	426 (27.9)	817 (26.8)	.431
Sleep duration, h	7.0 (6.0–8.0)	7.5 (6.5–8.0)	7.0 (6.0–8.0)	<.001
Use of sleeping medication^a^	60 (1.3)	18 (1.2)	42 (1.4)	.589
Comorbidity				
Hypertension	1884 (41.2)	456 (29.9)	1428 (46.8)	<.001
Diabetes	687 (15.0)	173 (11.3)	514 (16.8)	<.001
Dyslipidemia	3146 (68.7)	907 (59.4)	2239 (73.4)	<.001
Obesity	503 (11.0)	48 (3.2)	455 (14.9)	<.001
Chronic renal disease	105 (2.3)	42 (2.8)	63 (2.1)	.003
HIV-specific factors				
Y since HIV diagnosis		1.0 (0.2–4.4)		
Ever Efavirenz use^b^		772 (73.5)		
Nadir CD4+ T-cell counts, cells/µL		194.0 (106.0–288.0)		
Current CD4+ T-cell counts, cells/µL		352.0 (215.0–519.0)		
Current CD8+ T-cell counts, cells/µL		759.0 (529.0–1077.0)		
Current HIV viral suppression		1362 (89.3)		

Data are median (IQR) or No. (%).

Abbreviations: DBP, diastolic blood pressure; HDL-C, high-density lipoprotein cholesterol; IQR, interquartile range; LDL-C, low-density lipoprotein cholesterol; SBP, systolic blood pressure.

^a^Six observations were missing.

^b^Four hundred seventy-five observations were missing.

### Sleep Disorders and Physical Frailty in HIV-Positive Participants

Insomnia and poor sleep quality symptoms increased risk of physical frailty among middle-aged and older PWH in the crude models (Table 2). These results were also sustained after adjusting for age, gender, smoking, alcohol consumption, depression, neurocognitive impairment, comorbidities, and current CD4+ and CD8+ T-cell counts. The ORs of insomnia and poor sleep quality for physical frailty were 3.05 (95% CI, 1.63–5.72) and 2.32 (95% CI, 1.21–4.45), respectively. As for sleep duration, we found that both the short (<6 hours; OR, 4.27; 95% CI, 1.58–11.55) and long (≥9 hours; OR, 3.80; 95% CI, 1.33–10.86) sleep duration groups had significantly higher odds ratios for frailty than the reference group, after adjusting for potential confounders. Furthermore, stratified analysis observed that sleep disorders (insomnia: OR, 5.10; 95% CI, 2.21–11.79; poor sleep quality: OR, 4.79; 95% CI, 1.95–11.75; short sleep: OR, 5.43; 95% CI, 1.51–19.51; and long sleep duration: OR, 4.10; 95% CI, 1.04–16.08) were significantly associated with physical frailty in middle-aged and older PWH with current CD4+ T-cell counts <350 cells/µL, but not in those with CD4+ T-cell counts ≥350 cells/µL (Supplementary Table 1).

**Table 2.** Association of Insomnia, Sleep Quality, and Sleep Duration With Risk of Physical Frailty by HIV Serostatus

Sleep Variables	Model 1		Model 2		Model 3	
	OR (95% CI)	*P* Value	OR (95% CI)	*P* Value	OR (95% CI)	*P* Value
HIV-positive participants						
Insomnia						
No	Reference		Reference		Reference	
Yes	3.59 (2.16–5.95)	<.001	2.72 (1.59–4.67)	<.001	3.05 (1.63–5.72)	<.001
Poor sleep quality						
PSQI ≤5	Reference		Reference		Reference	
PSQI >5	3.31 (1.99–5.48)	<.001	2.10 (1.20–3.70)	.010	2.32 (1.21–4.45)	.011
Sleep duration						
<6 h	3.96 (1.61–9.74)	.003	2.98 (1.18–7.54)	.021	4.27 (1.58–11.55)	.004
≥6, <7 h	2.64 (1.06–6.56)	.037	2.32 (0.91–5.92)	.078	2.04 (0.69–5.99)	.196
≥7, <8 h	Reference		Reference		Reference	
≥8, <9 h	1.60 (0.68–3.78)	.284	1.85 (0.76–4.47)	.174	1.69 (0.62–4.61)	.303
≥9 h	3.03 (1.27–7.26)	.013	3.15 (1.24–7.97)	.016	3.80 (1.33–10.86)	.013
HIV-negative participants						
Insomnia						
No	Reference		Reference		Reference	
Yes	2.28 (1.50–3.48)	<.001	1.27 (0.78–2.06)	.335	1.30 (0.80–2.11)	.291
Poor sleep quality						
PSQI ≤5	Reference		Reference		Reference	
PSQI >5	2.48 (1.63–3.76)	<.001	1.32 (0.81–2.16)	.273	1.35 (0.82–2.22)	.235
Sleep duration						
<6 h	2.88 (1.54–5.39)	.001	1.57 (0.80–3.12)	.193	1.59 (0.80–3.14)	.188
≥6, <7 h	1.43 (0.73–2.80)	.296	1.27 (0.63–2.56)	.512	1.25 (0.62–2.54)	.531
≥7, <8 h	Reference		Reference		Reference	
≥8, <9 h	1.07 (0.52–2.20)	.857	0.96 (0.45–2.06)	.919	0.97 (0.45–2.07)	.928
≥9 h	5.13 (2.69–9.78)	<.001	3.32 (1.65–6.67)	<.001	3.24 (1.60–6.56)	.001

Model 1: unadjusted.

Model 2: adjusted for age, gender, smoking, alcohol consumption, depression, and neurocognitive impairment.

Model 3: adjusted for age, gender, smoking, alcohol consumption, depression, neurocognitive impairment, and comorbidity. HIV-positive participants were further adjusted for current CD4+ and CD8+ T-cell counts.

Abbreviations: OR, odds ratio; PSQI, The Pittsburgh Sleep Quality Index.

**Figure 2.**
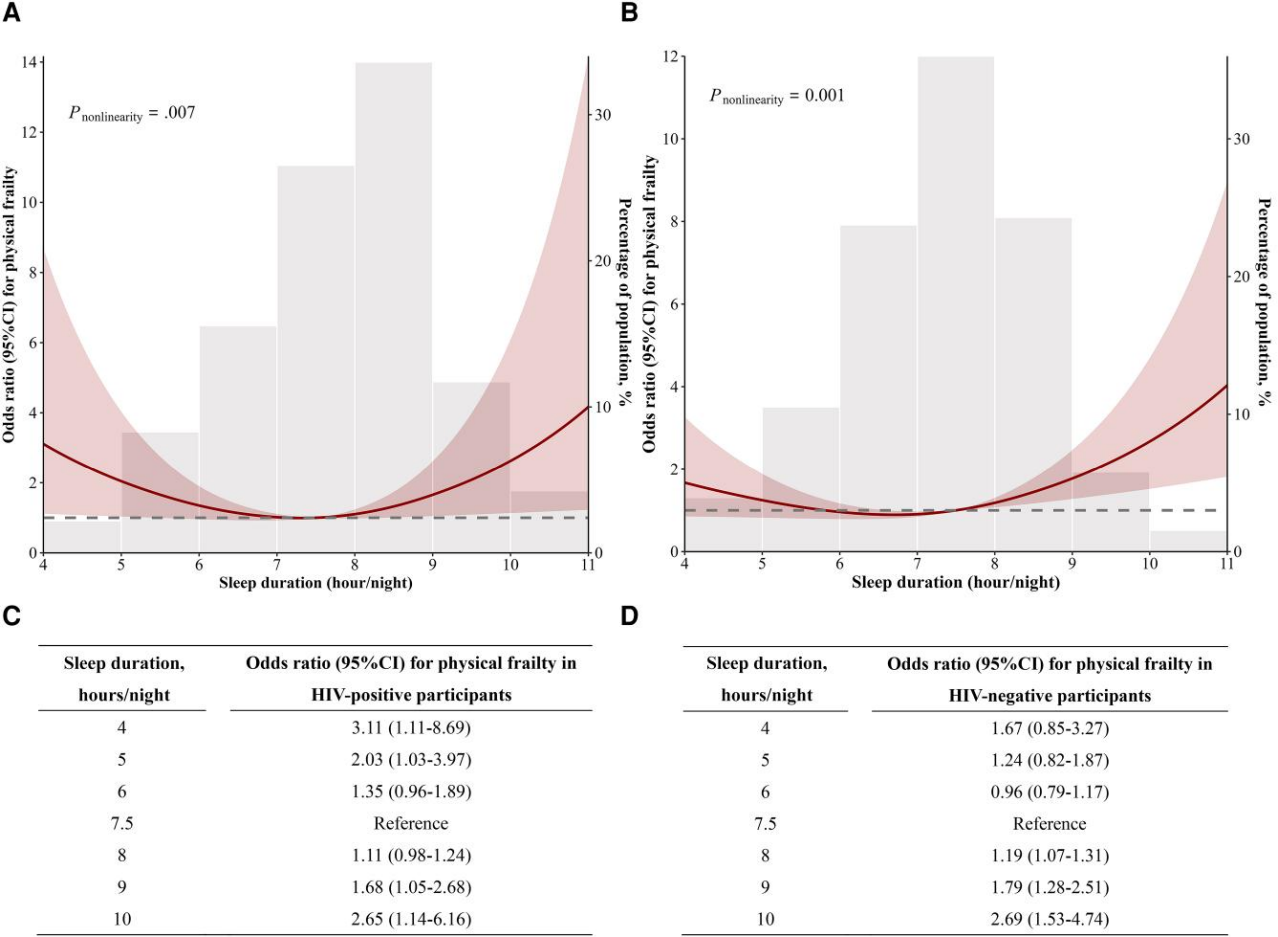
Associations of sleep duration with risk of physical frailty using a restricted cubic splines regression in HIV-positive (*A and C*) and HIV-negative (*B and D*) participants. Solid lines represent the odds ratios of physical frailty, adjusted for age, gender, smoking, alcohol consumption, depression, neurocognitive impairment, and comorbidity. HIV-positive participants were further adjusted for current CD4+ and CD8+ T-cell counts. The shaded areas represent the 95% CI. The histogram represents the distribution of study participants.

Results of logistic regression models with restricted cubic splines showed there was a significant nonlinear dose–response relation between sleep duration and physical frailty among PWH after adjusting for age, gender, smoking, alcohol consumption, depression, neurocognitive impairment, comorbidities, and current CD4+ and CD8+ T-cell counts (*P*_nonlinearity_ = .007) (Figure 2*A*). A U-shaped association was observed between sleep duration and frailty; either shorter or longer sleep duration was associated with an increased risk of frailty. For PWH with 4, 5, 9, and 10 hours/night of sleep duration, the ORs for frailty were 3.11 (95% CI, 1.11–8.69), 2.03 (95% CI, 1.03–3.97), 1.68 (95% CI, 1.05–2.68), and 2.65 (95% CI, 1.14–6.16), respectively (Figure 2*A*).

Mediation analysis observed that the association of insomnia, sleep quality, and sleep duration with risk of physical frailty was significantly mediated by depression among PWH (Table 3). After adjusting for potential confounding factors, depression partially mediated the association between insomnia and physical frailty, with a significant indirect effect (OR, 1.022; 95% CI, 1.011–1.037; *P* < .001) and a direct effect (OR, 1.036; 95% CI, 1.006–1.070; *P* = .024). In addition, depression completely mediated the association of poor sleep quality and sleep duration with frailty, which indicated a significant indirect effect (poor sleep quality: OR, 1.031; 95% CI, 1.017–1.049; *P* < .001; sleep duration: OR, 0.991; 95% CI, 0.987–0.995; *P* < .001) but a nonsignificant direct effect (sleep quality: OR, 1.021; 95% CI, 0.992–1.051; *P* = .158; sleep duration: OR, 1.005; 95% CI, 0.995–1.016; *P* = .294).

**Table 3.** Mediation Analysis of the Association of Sleep Disorders With Physical Frailty by Depression

Sleep Disorders	Coefficient (95% BCa CI)	OR (95% CI)	Z	*P* Value
HIV-positive participants				
Insomnia				
Indirect effect–path ab	0.022 (0.011–0.036)	1.022 (1.011–1.037)	3.655	<.001
Direct effect–path c'	0.035 (0.006–0.068)	1.036 (1.006–1.070)	2.262	.024
Total effect–path c	0.058 (0.028–0.090)	1.060 (1.028–1.094)	3.684	<.001
Poor sleep quality				
Indirect effect–path ab	0.031 (0.017–0.048)	1.031 (1.017–1.049)	3.945	<.001
Direct effect–path c'	0.021 (−0.008 to 0.050)	1.021 (0.992–1.051)	1.413	.158
Total effect–path c	0.051 (0.022–0.083)	1.052 (1.022–1.087)	3.388	<.001
Sleep duration				
Indirect effect–path ab	−0.009 (−0.013 to −0.005)	0.991 (0.987–0.995)	−4.521	<.001
Direct effect–path c'	0.005 (−0.005 to 0.016)	1.005 (0.995–1.016)	1.048	.294
Total effect–path c	−0.004 (−0.014 to 0.006)	0.996 (0.986–1.006)	−0.726	.468
HIV-negative participants				
Sleep duration				
Indirect effect–path ab	−0.004 (−0.006 to −0.002)	0.996 (0.994–0.998)	−4.194	<.001
Direct effect–path c'	0.005 (−0.001 to 0.011)	1.005 (0.999–1.011)	1.504	.132
Total effect–path c	0.001 (−0.005 to 0.007)	1.001 (0.995–1.007)	0.351	.726

Adjusted for age, gender, smoking, alcohol consumption, neurocognitive impairment, and comorbidity. HIV-positive participants were further adjusted for current CD4+ and CD8+ T-cell counts.

Path ab coefficient represents 5000 bootstrapped samples and BCa 95% CIs. Path c': the direct effect of the exposure variable (sleep disorders) on the outcome (physical frailty), holding the effect of depression constant. Path c: the total effect of exposure (sleep disorders) on the outcome (physical frailty).

Abbreviations: BCa, bias-corrected and accelerated; OR, odds ratio.

### Sleep Disorders and Physical Frailty in HIV-Negative Participants

As shown in Table 2, middle-aged and older HIV-negative individuals with symptoms of insomnia and poor sleep quality had an increased risk of physical frailty in the crude models, compared with those without insomnia and poor sleep quality. However, these results were nonsignificant after adjusting age, gender, smoking, alcohol consumption, depression, neurocognitive impairment, and comorbidities. The adjusted ORs of insomnia and poor sleep quality for frailty were 1.30 (95% CI, 0.80–2.11) and 1.35 (95% CI, 0.82–2.22), respectively. As for sleep duration, logistic regression models revealed a significantly increased risk of physical frailty for HIV-negative individuals with long sleep duration (≥9 hours) but not for those with short sleep duration (<6 hours), after adjusting for potential confounders. Compared with the sleep duration of 7–8 hours/night group, the adjusted ORs of short and long sleep duration on frailty in Model 3 were 1.59 (95% CI, 0.80–3.14) and 3.24 (95% CI, 1.60–6.56), respectively.

The results of logistic regression models with restricted cubic splines showed a significant nonlinear dose–response relation between sleep duration and physical frailty among middle-aged and older HIV-negative participants after adjusting for age, gender, smoking, alcohol consumption, depression, neurocognitive impairment, and comorbidity (*P*_nonlinearity_ = .001) (Figure 2*B*). A J-shaped association was observed, and longer sleep duration was associated with an increased risk of frailty. For HIV-negative participants with a sleep duration of 9 and 10 hours/night, the ORs for physical frailty were 1.79 (95% CI, 1.28–2.51) and 2.69 (95% CI, 1.53–4.74), respectively (Figure 2*B*).

Based on the significant association between sleep duration and physical frailty observed among HIV-negative individuals in the abovementioned results, a mediation analysis was further conducted to examine whether depression mediated the association between sleep duration and frailty. The results showed that the association between sleep duration and risk of frailty was significantly mediated by depression among HIV-negative participants. After adjusting for potential confounding factors, depression completely mediated the sleep duration–physical frailty association, with a significant indirect effect (OR, 0.996; 95% CI, 0.994–0.998; *P* < .001) but a nonsignificant direct effect (OR, 1.005; 95% CI, 0.999–1.011; *P* = .132) (Table 3).

## DISCUSSION

As far as we know, this was the first study to examine the association of insomnia, sleep quality, and sleep duration with risk of physical frailty among age/sex-matched middle-aged and older HIV-positive and HIV-negative participants. Our findings demonstrated that PWH experienced frailty at an earlier age and had a higher prevalence of frailty than negative controls. Insomnia and poor sleep quality increased the risk of physical frailty in PWH, but not in HIV-negative individuals. In addition, there was a U-shaped and J-shaped nonlinear dose–response relationship between sleep duration and frailty among HIV-positive and HIV-negative participants, respectively. The mediation analysis revealed that the relationships of insomnia, poor sleep quality, and sleep duration with physical frailty were mediated by depression in PWH.

In the present study, insomnia and poor sleep quality were significantly associated with physical frailty in middle-aged and older HIV-positive participants, but not in HIV-negative participants. In contrast to our findings, a previous cross-sectional study conducted in China, which included 345 HIV-infected and 345 HIV-uninfected participants aged ≥40 years, found no association between insomnia symptoms and frailty/prefrailty in middle-aged and older HIV-infected individuals. However, this study reported results consistent with ours in the general population, where insomnia was not found to be associated with frailty/prefrailty in HIV-uninfected individuals [29]. The exact relationship of insomnia and poor sleep quality with physical frailty in HIV-positive individuals is not fully understood due to a lack of research, and further studies are needed to explore this relationship in more detail.

Our stratified analysis revealed a significant association between sleep disorders (including insomnia, poor sleep quality, short sleep duration, and long sleep duration) and physical frailty in PWH with current CD4+ T-cell counts <350 cells/µL, but not in those with CD4+ T-cell counts ≥350 cells/µL. This discrepancy could be explained by differences in immune function and inflammation between the 2 groups. PWH with a CD4+ T-cell count <350 cells/µL may have a compromised immune system and are more susceptible to inflammation and infection, which may contribute to the development of physical frailty [30]. Poor sleep quality has been linked to increased inflammation and immune dysfunction in PWH, and chronic inflammation may further contribute to physical frailty [7, 30].

We observed a U-shaped dose–response relationship between sleep duration and physical frailty in HIV-positive participants, with both shorter and longer sleep duration associated with an increased risk of physical frailty. However, in HIV-negative participants, a J-shaped association was observed, with only longer but not shorter sleep duration associated with physical frailty. A previous study conducted among community-dwelling older adults in the United States reported results consistent with our findings in an HIV-negative population. They found that those who slept longer (≥10 hours) rather than shorter (≤6 hours) durations had double the possibility of being frail (OR, 2.86; 95% CI, 1.09–7.50) [31]. Similarly, studies conducted among Chinese [32] and Korean [33] older adults also found that long sleep duration was related to a higher risk of physical frailty. However, we were unable to compare our results regarding the effect of sleep duration on physical frailty among HIV-positive patients, as no similar study has been found on the association between sleep duration and frailty in PWH. More research is needed to fully elucidate the sleep duration–frailty association and develop targeted interventions to prevent and treat physical frailty in PWH with abnormal sleep duration.

The potential mechanisms underlying the associations between sleep disorders and physical frailty among HIV-positive patients are poorly understood. The mediation analysis used in this study may provide epidemiological evidence for a potential underlying mechanism of sleep disorders that may be associated with physical frailty in PWH through the mediation effect of depression. Sleep disorders, such as insomnia and poor sleep quality, are often comorbid with depression in HIV-positive patients. The previous literature has suggested that sleep disorders may lead to depression and decreased physical inactivity [34, 35], increasing the risk of negative health outcomes. Moreover, depression has been associated with dysregulation of the immune system, resulting in increased production of pro-inflammatory cytokines [36, 37], which may promote risk of physical frailty. Another mechanism explaining the association between sleep disorders and frailty among PWH is chronic inflammation and medication side effects. Chronic inflammation is a hallmark of HIV infection and may contribute to the development of frailty, especially in PWH with lower CD4+ T-cell counts [30]. Disruption of circadian rhythm due to sleep disorders in PWH may lead to immune system dysregulation, resulting in elevated levels of inflammatory molecules, such as interleukin 6 and C-reactive protein, that have been associated with the risk of frailty [38, 39]. Moreover, some antiretroviral medications, such as efavirenz, used to treat HIV can cause sleep disturbances. Several studies have reported the negative impact of antiretroviral medication side effects on sleep disorders in PWH. These medication-related sleep disturbances could exacerbate the risk of frailty in this population [40, 41].

Our findings may have important public health and clinical implications. Owing to widespread use of potent ART, growing numbers of PWH are living into older age. PWH experience an excess burden of age-related diseases earlier in life, including cognitive impairment [42] and geriatric syndromes such as frailty [23]. Previous studies have reported that PWH experience an earlier onset of physical frailty compared with HIV-negative controls with similar demographic characteristics and described a higher prevalence of physical frailty, which was found to be associated with adverse health outcomes, such as falls, disability, hospitalization, and death [3, 23]. Our study found that sleep disorders (insomnia, poor sleep quality, short or long sleep duration) were a risk factor for physical frailty and that appropriate interventions may improve this factor. Thus, clinicians caring for middle-aged and older HIV-positive patients need to be aware of the risk factor for frailty, identify sleep disorders and depression early, and intervene promptly to delay the onset and progression of physical frailty.

A major strength of our study was the large sample of HIV-positive patients and age/sex-matched HIV-negative controls. Moreover, we comprehensively explored the association of sleep disorders including insomnia, sleep quality, and sleep duration with physical frailty and further fitted a dose–response relation between sleep duration and frailty. In addition, we assessed the mediating effect of depression in the association between sleep and frailty, which could provide epidemiology evidence for the potential mechanism of the association. Nonetheless, there are some limitations to the present study. First, this was a cross-sectional study, and a causal relationship between sleep disorders and physical frailty could not be concluded. Second, sleep disorders were self-reported by study participants, and thus misreporting cannot be ruled out. The gold standard of actigraphy and polysomnography may provide more objective measures. However, polysomnography may not be feasible in a large-scale study due to its high expense and difficulty of use. Third, the overall duration of HIV since diagnosis was relatively short in our study, so our results should be interpreted with caution in other populations with a longer history of HIV diagnosis. More studies are needed to confirm these findings. Fourth, antidepressants and other psychotropic drugs may modify the sleep–frailty association. However, we were unable to consider their impact because this information was not collected. Finally, the participants in this study were all from eastern China, and therefore the generalizability of the findings may be limited.

## CONCLUSIONS

In summary, this study revealed that sleep disorders including insomnia, poor sleep quality, and short and long sleep duration were significantly associated with physical frailty among middle-aged and older HIV-positive patients, and depression was a mediator of this association. Although future longitudinal studies are necessary to further confirm these findings, our results emphasize that early detection of sleep disorders and psychological disturbances by regular monitoring and timely intervention would be helpful for preventing or delaying physical frailty progression in middle-aged and older PWH.

## Supplementary Material

ofad566_Supplementary_Data

## Supplementary Data

Supplementary materials are available at *Open Forum Infectious Diseases* online. Consisting of data provided by the authors to benefit the reader, the posted materials are not copyedited and are the sole responsibility of the authors, so questions or comments should be addressed to the corresponding author.

## References

[1] Wada N , Jacobson LP, Cohen M, French A, Phair J, Munoz A. Cause-specific mortality among HIV-infected individuals, by CD4(+) R cell count at HAART initiation, compared with HIV-uninfected individuals. AIDS 2014; 28:257–65. 24105030 10.1097/QAD.0000000000000078 PMC4164055

[2] May M , Gompels M, Delpech V, et al Impact of late diagnosis and treatment on life expectancy in people with HIV-1: UK Collaborative HIV Cohort (UK CHIC) study. BMJ 2011; 343:d6016. 21990260 10.1136/bmj.d6016 PMC3191202

[3] Zhou QG , He JY, Yang X, Yin H, Zhang ZY, He N. The association between physical frailty and injurious falls and all-cause mortality as negative health outcomes in people living with HIV: a systematic review and meta-analysis. Int J Infect Dis 2023; 126:193–9. 36455810 10.1016/j.ijid.2022.11.030

[4] Tassiopoulos K , Abdo M, Wu KL, et al Frailty is strongly associated with increased risk of recurrent falls among older HIV-infected adults. AIDS 2017; 31:2287–94. 28991026 10.1097/QAD.0000000000001613 PMC5654616

[5] Akgun KM , Tate JP, Crothers K, et al An adapted frailty-related phenotype and the VACS index as predictors of hospitalization and mortality in HIV-infected and uninfected individuals. J Acquir Immune Defic Syndr 2014; 67:397–404. 25202921 10.1097/QAI.0000000000000341 PMC4213242

[6] Ning C , Lin H, Chen X, et al Cross-sectional comparison of various sleep disturbances among sex- and age-matched HIV-infected versus HIV-uninfected individuals in China. Sleep Med 2020; 65:18–25. 31706188 10.1016/j.sleep.2019.06.020

[7] Wu J , Wu H, Lu CY, Guo L, Li PS. Self-reported sleep disturbances in HIV-infected people: a meta-analysis of prevalence and moderators. Sleep Med 2015; 16:901–7. 26188954 10.1016/j.sleep.2015.03.027

[8] Bao YP , Han Y, Ma J, et al Cooccurrence and bidirectional prediction of sleep disturbances and depression in older adults: meta-analysis and systematic review. Neurosci Biobehav R 2017; 75:257–73. 10.1016/j.neubiorev.2017.01.032 28179129

[9] Moreno-Tamayo K , Manrique-Espinoza B, Ortiz-Barrios LB, Cardenas-Bahena A, Ramirez-Garcia E, Sanchez-Garcia S. Insomnia, low sleep quality, and sleeping little are associated with frailty in Mexican women. Maturitas 2020; 136:7–12. 32386668 10.1016/j.maturitas.2020.03.005

[10] Zhao Y , Lu Y, Zhao W, et al Long sleep duration is associated with cognitive frailty among older community-dwelling adults: results from West China health and aging trend study. BMC Geriatr 2021; 21:608. 34706663 10.1186/s12877-021-02455-9 PMC8555015

[11] Liu S , Hu Z, Guo Y, Zhou F, Li S, Xu H. Association of sleep quality and nap duration with cognitive frailty among older adults living in nursing homes. Front Public Health 2022; 10:963105. 36091504 10.3389/fpubh.2022.963105 PMC9453392

[12] Liu XY , Wang CL, Qiao XX, Si HX, Jin YR. Sleep quality, depression and frailty among Chinese community-dwelling older adults. Geriatr Nurs 2021; 42:714–20. 33836251 10.1016/j.gerinurse.2021.02.020

[13] Lin HJ , Ding YY, Ning CX, et al Age-specific associations between HIV infection and carotid artery intima-media thickness in China: a cross-sectional evaluation of baseline data from the CHART cohort. Lancet Hiv 2019; 6:E860–8. 31635991 10.1016/S2352-3018(19)30263-2

[14] Zung WW . A self-rating depression scale. Arch Gen Psychiatry 1965; 12:63–70. 14221692 10.1001/archpsyc.1965.01720310065008

[15] Lombardi D , Mizuno LT, Thornberry A. The use of the zung self-rating depression scale to assist in the case management of patients living with HIV/AIDS. Care Manag J 2010; 11:210–6. 21197926 10.1891/1521-0987.11.4.210

[16] Yuan S , Chen X, Lin H, et al Interaction of declined handgrip strength and HIV infection on neurocognitive impairment. J Neurovirol 2022; 28:217–24. 34873670 10.1007/s13365-021-01036-1

[17] Zhou BF ; Cooperative meta-analysis group of the China Obesity Task Force. Predictive values of body mass index and waist circumference for risk factors of certain related diseases in Chinese adults—study on optimal cut-off points of body mass index and waist circumference in Chinese adults [in Chinese]. Biomed Environ Sci 2002; 15:83–96. 12046553

[18] Buysse DJ , Reynolds CF III, Monk TH, Berman SR, Kupfer DJ. The Pittsburgh Sleep Quality Index: a new instrument for psychiatric practice and research. Psychiatry Res 1989; 28:193–213. 2748771 10.1016/0165-1781(89)90047-4

[19] Hirshkowitz M , Whiton K, Albert SM, et al National sleep Foundation's sleep time duration recommendations: methodology and results summary. Sleep Health 2015; 1:40–3. 29073412 10.1016/j.sleh.2014.12.010

[20] Castro-Costa E , Dewey ME, Ferri CP, et al Association between sleep duration and all-cause mortality in old age: 9-year follow-up of the Bambui Cohort Study, Brazil. J Sleep Res 2011; 20:303–10. 20860564 10.1111/j.1365-2869.2010.00884.x

[21] Jenkins CD , Stanton BA, Niemcryk SJ, Rose RM. A scale for the estimation of sleep problems in clinical research. J Clin Epidemiol 1988; 41:313–21. 3351539 10.1016/0895-4356(88)90138-2

[22] Fried LP , Tangen CM, Walston J, et al Frailty in older adults: evidence for a phenotype. J Gerontol A Biol Sci Med Sci 2001; 56:M146–56. 11253156 10.1093/gerona/56.3.m146

[23] Li J , Chen X, Lin H, et al Associations between HIV infection and frailty status and its individual components: are frailty components disproportionally affected? HIV Med 2023; 24:533–43. 36288971 10.1111/hiv.13429

[24] Song JY , Cheong HJ, Noh JY, Choi MJ, Yoon JG, Kim WJ. Immunogenicity and safety of 13-valent pneumococcal conjugate vaccine in HIV-infected adults in the era of highly active antiretroviral therapy: analysis stratified by CD4 T-cell count. Hum Vaccin Immunother 2020; 16:169–75. 31441710 10.1080/21645515.2019.1643677 PMC7012181

[25] Hayes AF . Introduction to Mediation, Moderation, and Conditional Process Analysis: A Regression-Based Approach. Guilford Publications; 2022.

[26] Igartua JJ , Hayes AF. Mediation, moderation, and conditional process analysis: concepts, computations, and some common confusions. Span J Psychol 2021; 24:e49. 35923144 10.1017/SJP.2021.46

[27] Zhao X , Lynch JG Jr, Chen Q. Reconsidering Baron and Kenny: myths and truths about mediation analysis. J Consum Res 2010; 37:197–206.

[28] Joundi RA , O'Connell ME, Patten S, Smith EE. Mediation of post-stroke function by cognition in the Canadian Longitudinal Study on Aging. Can J Neurol Sci 2023: 1–9. 10.1017/cjn.2023.6 36627236

[29] Ding Y , Lin H, Liu X, et al Higher prevalence of frailty among a sample of HIV-infected middle-aged and older Chinese adults is associated with neurocognitive impairment and depressive symptoms. J Infect Dis 2017; 215:687–92. 28329145 10.1093/infdis/jix032

[30] Derry HM , Johnston CD, Burchett CO, et al Links between inflammation, mood, and physical function among older adults with HIV. J Gerontol B Psychol Sci Soc Sci 2022; 77:50–60. 33580236 10.1093/geronb/gbab027 PMC8755907

[31] Baniak LM , Yang K, Choi J, Chasens ER. Long sleep duration is associated with increased frailty risk in older community-dwelling adults. J Aging Health 2020; 32:42–51. 30270714 10.1177/0898264318803470 PMC6440876

[32] Sun XH , Ma T, Yao S, et al Associations of sleep quality and sleep duration with frailty and pre-frailty in an elderly population Rugao Longevity and Ageing Study. BMC Geriatr 2020; 20:9. 10.1186/s12877-019-1407-5 PMC6945401 31906855

[33] Kang I , KimS, KimBS, YooJ, KimM, WonCW. Sleep latency in men and sleep duration in women can be frailty markers in community-dwelling older adults: the Korean Frailty and Aging Cohort Study (KFACS). J Nutr Health Aging2019; 23:63–7.3056907010.1007/s12603-018-1109-2

[34] Saberi P , Neilands TB, Johnson MO. Quality of sleep: associations with antiretroviral nonadherence. AIDS Patient Care STDS 2011; 25:517–24. 21770763 10.1089/apc.2010.0375 PMC3157301

[35] Magee CA , Huang XF, Iverson DC, Caputi P. Examining the pathways linking chronic sleep restriction to obesity. J Obes 2010; 2010:821710. 10.1155/2010/821710 PMC2925323 20798899

[36] Poudel-Tandukar K , Bertone-Johnson ER, Palmer PH, Poudel KC. C-reactive protein and depression in persons with human immunodeficiency virus infection: the Positive Living With HIV (POLH) study. Brain Behav Immun 2014; 42:89–95. 24929193 10.1016/j.bbi.2014.06.004

[37] Ford DE , Erlinger TP. Depression and C-reactive protein in US adults: data from the Third National Health and Nutrition Examination Survey. Arch Intern Med 2004; 164:1010–4. 15136311 10.1001/archinte.164.9.1010

[38] Wirth MD , Jaggers JR, Dudgeon WD, et al Association of markers of inflammation with sleep and physical activity among people living with HIV or AIDS. AIDS Behav 2015; 19:1098–107. 25399034 10.1007/s10461-014-0949-y PMC4433614

[39] Erlandson KM , Ng DK, Jacobson LP, et al Inflammation, immune activation, immunosenescence, and hormonal biomarkers in the frailty-related phenotype of men with or at risk for HIV infection. J Infect Dis 2017; 215:228–37. 27799351 10.1093/infdis/jiw523 PMC5897840

[40] Nunez M , Gonzalez de Requena D, Gallego L, Jimenez-Nacher I, Gonzalez-Lahoz J, Soriano V. Higher efavirenz plasma levels correlate with development of insomnia. J Acquir Immune Defic Syndr 2001; 28:399–400. 10.1097/00126334-200112010-00015 11707679

[41] Lochet P , Peyriere H, Lotthe A, Mauboussin JM, Delmas B, Reynes J. Long-term assessment of neuropsychiatric adverse reactions associated with efavirenz. HIV Med 2003; 4:62–6. 12534961 10.1046/j.1468-1293.2003.00136.x

[42] Heaton RK , Clifford DB, Franklin DR Jr, et al HIV-associated neurocognitive disorders persist in the era of potent antiretroviral therapy: cHARTER study. Neurology 2010; 75:2087–96. 21135382 10.1212/WNL.0b013e318200d727 PMC2995535

