# Supplementary material for: Association of Insomnia, Sleep Quality, and Sleep Duration With Risk of Physical Frailty in Middle-aged and Older People With HIV
Source: Open Forum Infect Dis. 2023 Nov 9;10(11):ofad566. doi: 10.1093/ofid/ofad566 (PMC10686336; doi:10.1093/ofid/ofad566)
Supplement: ofad566_Supplementary_Data [file ofad566_supplementary_data.docx]

**Supplementary Materials**

**Table S1.** Association of sleep disorders with physical frailty in HIV-positive participants by current CD4+ T-cell counts

**Figure S1.** The simple mediation model of the association between sleep disorders and physical frailty by depression. Path c reflects the total effect, path c′ reflects the direct effect and path ab reflects the indirect effect.

Table S1 Association of sleep disorders with physical frailty in HIV-positive participants by current

CD4+ T-cell counts

| Sleep disorders | CD4+ T-cell counts≥350 | |  | CD4+ T-cell counts<350 | |
| --- | --- | --- | --- | --- | --- |
|  | OR (95% CI) | *P*-value |  | OR (95% CI) | *P*-value |
| Insomnia |  |  |  |  |  |
| No | Reference |  |  | Reference |  |
| Yes | 0.98 (0.30-3.23) | 0.969 |  | 5.10 (2.21-11.79) | <0.001 |
| Poor sleep quality |  |  |  |  |  |
| PSQI≤5 | Reference |  |  | Reference |  |
| PSQI>5 | 0.67 (0.21-2.17) | 0.501 |  | 4.79 (1.95-11.75) | <0.001 |
| Sleep duration |  |  |  |  |  |
| <6 hour | 1.93 (0.34-11.04) | 0.459 |  | 5.43 (1.51-19.51) | 0.010 |
| ≥6, <7 hour | 1.23 (0.18-8.41) | 0.836 |  | 2.33 (0.60-9.09) | 0.225 |
| ≥7, <8 hour | Reference |  |  | Reference |  |
| ≥8, <9 hour | 1.26 (0.25-6.33) | 0.779 |  | 1.72 (0.47-6.36) | 0.415 |
| ≥9 hour | 3.22 (0.58-17.88) | 0.181 |  | 4.10 (1.04-16.08) | 0.043 |

OR (95% CI) are adjusted for age, gender, smoking, alcohol drinking, depression, neurocognitive impairment, comorbidity,

current CD4+ and CD8+ T-cell counts. OR, odds ratios; CI, confidence interval; PSQI, The Pittsburgh Sleep Quality Index.


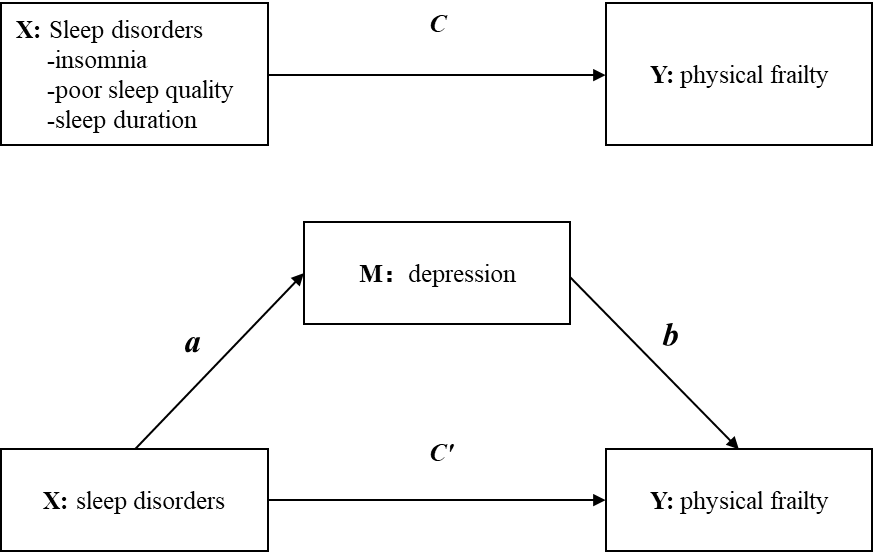


Figure S1 The simple mediation model of the association between sleep disorders and physical frailty by depression. Path c reflects the total effect, path c′ reflects the direct effect and path ab reflects the indirect effect.
